# Di‐*p*‐Coumaroyl Spermidine From Bee Pollen Alleviates Chronic Nonbacterial Prostatitis

**DOI:** 10.1002/fsn3.70467

**Published:** 2025-06-22

**Authors:** Jiawen Zhang, Jiangtao Qiao, Yu Zhang, Hequan Zhu, Eric Haubruge, Liqiang Liu, Jie Dong

**Affiliations:** ^1^ School of Basic Medical Sciences Capital Medical University Beijing China; ^2^ School of Life Sciences and Food Engineering Hebei University of Engineering Handan China; ^3^ Terra Research Center, Gembloux Agro‐Bio Tech University of Liege Gembloux Belgium; ^4^ State Key Laboratory of Resource Insects, Institute of Apicultural Research Chinese Academy of Agricultural Sciences Beijing China; ^5^ Jiangsu Beevip Biotechnology Co., Ltd Taizhou China

**Keywords:** AMPK/mTOR, anti‐inflammation, di‐*p*‐coumaroyl spermidine, phenolamides, rapeseed bee pollen

## Abstract

Bee pollen and its extracts have been used for decades as therapeutic agents or health food supplements to alleviate chronic nonbacterial prostatitis (CNP). However, the functional compounds in bee pollen that are anti‐CNP remain still unclear. In this study, we evaluated the anti‐CNP properties of six principal phenolamides in bee pollen. Our results provide compelling evidence that the anti‐CNP property of bee pollen may be ascribed to its abundance of phenolamides. Particularly, di‐*p*‐Coumaroyl spermidine can alleviate CNP by upregulating autophagy via the AMPK/mTOR signaling pathway and regulating gut microbiota, based on the cellular and rat models. Additionally, our finding may provide a novel insight into the gut–prostate axis by regulating di‐*p*‐Coumaroyl spermidine. This is the first report that di‐*p*‐Coumaroyl spermidine in bee pollen possesses the anti‐prostatitis function. This paper will likely be helpful further to develop functional foods, personalized nutraceuticals, and medicine from bee pollen.

## Graphical abstract

Di‐p‐Coumaroyl spermidine alleviates chronic non‐bacterial prostatitis by stimulating autophagy pathway and regulating gut microbiota.

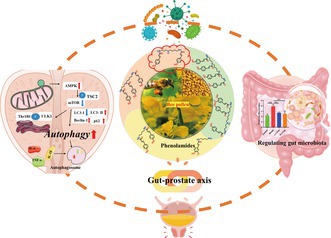

## 1 Introduction

Pollen, comprising the male gametophytic cells of angiosperms, is a finely powdered substance characterized by microscopic grains encapsulating the plant's male cells (Campos et al. 2008). Embedded within the realm of traditional medicine and functional food for centuries, bee pollen has been ascribed with manifold therapeutic properties, including anti‐inflammatory, antioxidant, antimicrobial, and immune‐enhancing attributes (Denisow and Denisow‐Pietrzyk 2016). In recent decades, there has been a renewed surge in the attention devoted to bee pollen, driven by its pronounced effectiveness in mitigating benign prostatic hyperplasia and chronic prostatitis (Algethami et al. 2022; Chabot et al. 2021; Qiao, Xiao, et al. 2023).

As the global population ages, the incidence of chronic prostatitis in men is increasing, with older age being a significant risk factor for the development of this condition. Chronic nonbacterial prostatitis (CNP) is clinically defined as a persistent inflammatory process leading to chronic pelvic pain or discomfort accompanied by urinary symptoms and sexual dysfunction (Zhang, Liang, et al. 2020). It has emerged as a significant male healthcare issue, attracting considerable attention from clinicians and researchers alike (Krieger et al. 2003). Notably, a clinical research study conducted across North America, Europe, and Asia revealed that the prevalence of chronic prostatitis ranges from 2% to 10% among adult males at any given time, while approximately 15% of men experience prostatitis symptoms during their lifetime (Krieger et al. 2003). Furthermore, previous investigations have indicated a potential link between chronic prostatitis and an increased risk for developing benign prostatic hyperplasia and prostate cancer (Zhang, Wang, et al. 2020). Fortunately, researchers have discovered that pollen or pollen extracts demonstrate notable efficacy in preventing and treating chronic prostatitis, leading to their widespread application worldwide (Algethami et al. 2022; Chabot et al. 2021; Lazzarotto‐Figueiró et al. 2020; Martinez‐Armenta et al. 2021; Münstedt and Bogdanov 2009; Nakase et al. 1990). In China, Pule'an capsules, which are made entirely from rapeseed bee pollen, have emerged as the prevailing pharmaceutical option for chronic prostatitis and prostate hyperplasia (Qiao, Xiao, et al. 2023). Clinical trials have shown that Pule'an capsules can effectively alleviate the symptoms of chronic prostatitis and prostate hyperplasia patients, with a symptom relief rate exceeding 90% (Qiao, Xiao, et al. 2023). Moreover, rapeseed bee pollen extracts have been discovered to substantially alleviate symptoms associated with CNP (Wagner et al. 2013; Yang et al. 2014). This effect is achieved through modulating some key molecular factors, including the downregulation of mitofusin‐1 (Mfn1) levels within the posterior lobes of the prostate, as well as the suppression of DHT, 5α‐reductase, and Cyclooxygenase‐2 (COX‐2) levels (Wagner et al. 2013; Yang et al. 2014). In Europe and Japan, Cernitin (Pharmaceutical Brand, Cernilton), ryegrass pollen extract, has been used to treat nonbacterial prostatitis and prostatic hyperplasia for more than 40 years with an overall success rate of 70% (Chung et al. 2021; Thorpe and Neal 2003). Cernitin can notably reduce the prostate‐specific antigen levels associated with chronic prostatitis/chronic pelvic pain syndrome. In preclinical studies, Cernitin demonstrated significant pain relief in an induced prostatitis rat model, concomitant with a noteworthy decrease in intraprostatic levels of COX‐2 and MCP‐1 (Chung et al. 2021; El‐Khatib et al. 2019; Thorpe and Neal 2003). Cernitin consists of two fractions of pollen extract: water‐soluble T60 and lipid‐soluble GBX (Chung et al. 2021; El‐Khatib et al. 2019; Thorpe and Neal 2003). The two fractions can distinctly reduce inflammation, inhibit cellular proliferation, and relax smooth muscle in animal models (Nakase et al. 1990). Evidence suggests that the anti‐CNP effect of the T60 fraction is attributed to feruloyl putrescine (Nakase et al. 1990).

Feruloyl putrescine is formed by ferulic acid and putrescine through an amide bond, a type of phenolamide. Phenolamides, also known as hydroxycinnamic acid amides, result from the conjugation of hydroxycinnamic acids (e.g., p‐coumaric, ferulic, and caffeic acid) with aliphatic or aromatic amines, such as putrescine, spermine, and spermidine. Our previous study has shown that bee pollen is a treasure trove of phenolamides (Qiao, Feng, et al. 2023). Sixty‐four phenolamides were identified in 20 types of monofloral bee pollen, with the highest contents among known natural products (Qiao, Feng, et al. 2023). Moreover, in the last 5 years, multiple studies have reported the presence of abundant phenolamides in bee pollen (Kim et al. 2018; Zhang, Zhu, et al. 2023; Zhang, Wu, et al. 2023). Hence, the robust inhibitory functionality of bee pollen or extracts against prostatitis may be attributed to its abundant phenolamides. However, apart from feruloyl putrescine, the anti‐CNP activities of other phenolamides from bee pollen remained unclear.

The current study aimed to evaluate the anti‐CNP effects and mechanisms of phenolamides in bee pollen. Firstly, we employed an LPS‐induced prostate epithelial cell (RWPE‐1) model to screen anti‐prostatitis activities from six representative phenolamides in bee pollen. Subsequently, we elucidated anti‐prostatitis mechanisms using a CNP rat model.

## 2 Materials and Methods

### 2.1 Materials

**FIGURE 1:**
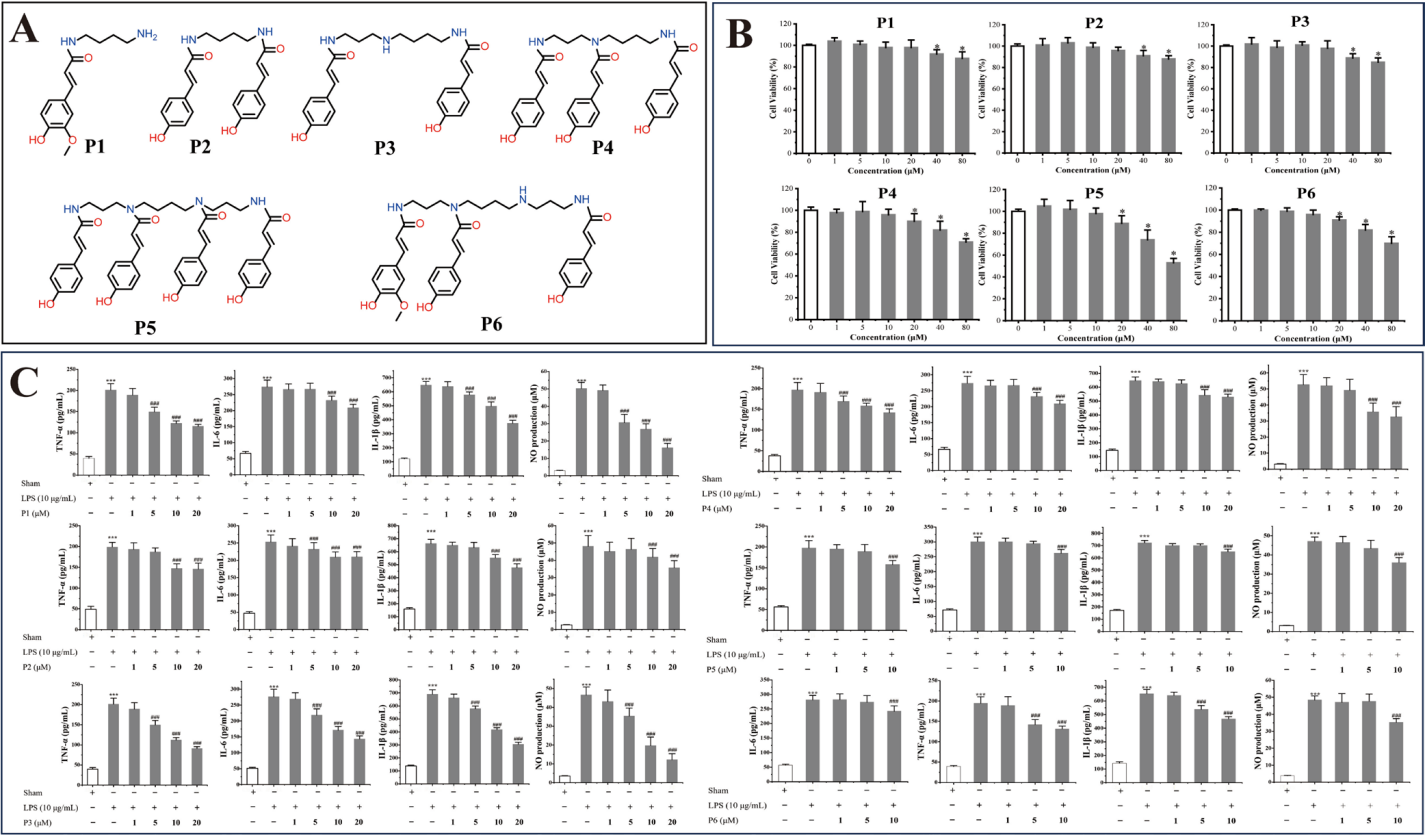
The six phenolamides in bee pollen exert inhibitory effects on LPS‐induced inflammation in RWPE‐1 cells. (A) Six phenolamides in bee pollen; P1, N(E)‐feruloyl putrescine; P2, N(E), N′ (E)‐di‐*p*‐coumaroyl putrescine; P3, N1(E), N10(E)‐di‐*p*‐coumaroyl spermidine; P4, N1(E), N5(E), N10(E)‐tri‐*p*‐coumaroyl spermidine; P5, N1(E), N5(E), N10(E), N14(E)‐tetra‐ *p*‐coumaroyl spermine; P6, N1(E), N10(E)‐di‐*p*‐coumaroyl‐N14(E)‐ feruloyl spermine. (B) Effect of six phenolamides on cellular viability by MTT assay. *p < 0.05. (C) Effect of six phenolamides on proinflammatory cytokines and NO production in LPS‐induced RWPE‐1 cells. ***p < 0.001 vs Sham group, ### *p* < 0.001 vs LPS.

N(E), N' (E)‐di‐*p*‐coumaroyl putrescine, N1(E), N10(E)‐di‐*p*‐coumaroyl spermidine, N1(E), N5(E), N10(E)‐tri‐*p*‐coumaroyl spermidine, N1(E), N5(E), N10(E), N14(E)‐tetra‐ *p*‐coumaroyl spermine, and N1(E), N10(E)‐ di‐*p*‐coumaroyl‐N14(E)‐ feruloyl spermine were prepared (purity ≥ 95%) according to our previous study (Qiao, Feng, et al. 2023). N(E)‐feruloyl putrescine (purity ≥ 95%) was purchased from Shanghai Yuanye Biotechnology Co. Ltd. (Shanghai, China). All the chemical structures are shown in Figure 1A.

Human TNF‐α, IL‐1β, IL‐6 and Rat TNF‐α, IL‐1β, IL‐6 Kit purchased from Solarbio (Beijing, China). NO kits (Beyotime, Nanjing, China). Anti‐TNF‐alpha Rabbit pAb (Servicebio, GB11188), Anti‐IL‐1 beta Rabbit pAb (Servicebio, GB11113), Anti‐vIL‐6 Rabbit pAb (Servicebio, GB11117), Cy3 conjugated Goat Anti‐Rabbit IgG (H + L) (Servicebio, GB21303). Beclin‐1 pAb (YT5763), LC3 A/B Rabbit pAb (YT7936), GAPDH mAb (YM3029) purchased from ImmunoWay Biotechnology Company (TX,75024 USA). Rabbit anti‐p62 antibody (5114), p‐AMPK (2535), AMPK (5831), p‐mTOR (5536) and mTOR (2983) from Cell Signaling Technology (Danvers, MA, USA). Complete Freund's adjuvant (CFA) was purchased from Sinopharm Co. Ltd. (Shanghai, China). Cole's Hematoxylin solution, Eosin Y solution, and Ethylene Diamine Tetraacetic Acid (EDTA) pH 8.0 were obtained from Abcam (Cambridge, MA, USA). All other experimental materials and consumables were purchased from Solarbio Life Sciences (Beijing, China).

### 2.2 RWPE‐1 Cell Culture

The immortalized human prostate epithelial cell line (RWPE‐1) was purchased from BNCC (Henan, China). RWPE‐1 cells were cultured in keratinocyte medium (ScienceCell, San Diego, California, USA) supplemented with 5 mL of keratinocyte growth supplement (KGS, Cat#2152) and 5 mL of penicillin/streptomycin solution (P/S, Cat. #0503) in a humidified 37°C incubator with 5% CO_2_. The model of CNP was induced by 10 μg/mL lipopolysaccharide (LPS) for 24 h.

### 2.3 Cell Viability Assays

RWPE‐1 cell viability was evaluated using the methyl thiazolyl tetrazolium assay (MTT, Solarbio, Beijing, China). RWPE‐1 cells (20,000 cells/well) were seeded in a 96‐well plate and incubated at 37°C for 24 h. Subsequently, the culture medium was replaced respectively with 1 mL fresh keratinocyte medium containing 0.1% DMSO as a control group and 1 mL various concentrations of phenolamides (0, 1, 5, 10, 20, 40, and 80 μmol/L) as treatment groups. After the treatment, MTT solution (5 mg/mL) was added to each well and incubated for 4 h. The absorbance was measured at 490 nm using a microplate reader (Biotek, USA). All experiments were performed in triplicate. Cell viability was calculated as a percentage using the following formula: cell viability = [(mean absorbance of each treatment group) / (mean absorbance of the control group)] × 100%.

### 2.4 Inflammatory Cytokines and NO Production of RWPE‐1 Cells

RWPE‐1 cells were incubated in 24‐well culture plates and treated with different concentrations of phenolamides (0, 1, 5, 10, 20, 40, 80 μmol/L) for 2 h, and then the cells were stimulated with 5 μg/mL LPS and incubated for 24 h. TNF‐α, IL‐1β, and IL‐6 levels were quantified using a commercially available ELISA kit from Solarbio (Beijing, China). Based on Greiss reaction, the nitric oxide (NO) concentration in the cell culture media was determined using NO kits (Beyotime, Nanjing, China).

### 2.5 Animal Model

All animal experimental procedures followed the Guidelines for the Care and Use of Laboratory Animals. Fifty‐two‐month‐old male Sprague‐Dawley rats (190–220 g) were obtained from the specific pathogen‐free (SPF) biotechnology Co. Ltd., Beijing, China (licenses: 110324231104817715; animal use licenses: SYXK(JING) 2023‐0004). We kept all the animals under SPF environmental conditions at 23°C ± 2°C and relative humidity of 55% ± 5%. Besides, the rats had free access to food and water in a 12 h dark–light cycle (light cycle: 7:00 am–7:00 pm) and were given 1 week to adapt to the new environment before the experiment. The experimental Animal Welfare Committee of the Institute of Apicultural Research, Chinese Academy of Agricultural Sciences approved ethical requirements. Using a random number table, 36 rats were randomly divided into four groups (*n* = 9 rats/group), including the control group, model group, and N1(E), N10(E)‐di‐*p*‐coumaroyl spermidine (*p*‐Csp) low or high dose group. The sample size was determined based on our previous studies (Algethami et al. 2022; Chabot et al. 2021; Qiao, Xiao, et al. 2023).

The rat model of CNP was established using CFA as a previously described method with slight modification (Zhao et al. 2020). Apart from the control group, the intervention involved the injection of a sterile CFA suspension (100 μL, 1% w/v) into the right and left lobes of the ventral prostate; while for the sham‐operated rats (*n* = 9), the same volume of sterile saline was injected. The wound was then closed in layers. During surgical procedures, isoflurane inhalation anesthesia was used. Isoflurane was administered at an induction concentration of 4%–5% in 100% oxygen and maintained at 1.5%–2.5% during the procedure. The anesthetic gas mixture was delivered through a rodent‐specific nose cone to ensure the animals remained unconscious and pain‐free. The control group and model group were orally administered 0.5% DMSO (w/w) 1 mL for 21 days. The remaining two groups of rats were orally administered 1 mL *p*‐Csp dissolved in 0.5% DMSO (w/w), according to 5 mg/kg (low dosage group, L‐*p*‐Csp) and 10 mg/kg (low dosage group, H‐*p*‐Csp) body weight for 21 days. On the final day of the experiment (day 22), all rats were humanely euthanized. The euthanasia of animals (rats) was conducted using carbon dioxide (CO_2_) asphyxiation, following the AVMA Guidelines for the Euthanasia of Animals. CO_2_ was introduced gradually into the chamber at a displacement rate of 30%–70% of the chamber volume per minute to minimize distress, ensuring unconsciousness prior to respiratory arrest. Subsequently, the prostate tissues were carefully harvested and promptly weighed. The prostate index was then computed as the ratio of the prostate weight to the total body weight.

The order of treatments and measurements for each group was not predetermined and was conducted randomly each time. In addition, cage location was unintentionally assigned to available address upon the arrival of animals at the experimental facility.

### 2.6 Histopathology and Immunohistochemical

The prostate specimens were fixed in 4% formaldehyde overnight for histopathological examination. Fixed prostate specimens were embedded in paraffin and sectioned with a microtome (5 μm) using a Leica RM2255 Microtome (Leica Biosystems, Leica, Germany). After deparaffinization with xylene and dehydration using alcohol, the sections were subjected to hematoxylin–eosin staining. Subsequently, the glandular epithelium, structure, and space were meticulously examined using a Nikon Eclipse Ci‐L microscope equipped with a Nikon digital sight DS‐Fi2 system (Nikon, Tokyo, Japan). Histomorphometry parameters of the normal prostate were determined based on the analysis from the control group.

Slides containing 5 μm thick rat prostate sections were meticulously rinsed with phosphate‐buffered saline (PBS) and then permeabilized with 0.1% Triton X‐100 in PBS for 10 min, followed by blocking with 2% bovine serum albumin (BSA) in PBS for 30 min. The primary antibody was applied and left to incubate overnight at a temperature of 4°C. Subsequently, the slides were thoroughly washed with PBS and incubated with a secondary antibody at room temperature for 50 min. After another round of meticulous washing, the immunofluorescent images were captured using an 80i Nikon microscope. The images were captured and analyzed with Aipathwell software (Servicebio, Wuhan, China). The positive cell ratio was calculated to assay the expression of IL‐6, IL‐1β, and TNF‐α. Positive cells ratio = the number of positive cells/total number of cells. Moreover, a Nikon Eclipse Ti confocal laser scanning microscope with DS‐U3 imaging system (Nikon, Tokyo, Japan) to scan inflammatory cytokines for visualization.

### 2.7 Inflammatory Cytokines

In this study, we determined the levels of IL‐6, IL‐1β, and TNF‐α in both serum and prostate tissue using an ELISA kit. For serum analysis, each sample was meticulously collected in serum separator tubes (BD Biosciences, Rockville, USA) and allowed to clot for 2 h at room temperature. Following the clotting period, centrifugation was performed at approximately 1000 × g for 20 min to remove the clots and obtain the serum samples. As for the prostate tissue, a rigorous protocol was employed to ensure proper sample preparation. Each prostate tissue specimen was meticulously rinsed with ice‐cold PBS (0.01 M, pH = 7.4) to remove residual blood thoroughly. Subsequently, the prostate samples were weighed, and using an electric homogenizer, they were homogenized in ice‐cold PBS. The homogenates were centrifuged at 5000 × g for 5 min to obtain the supernatants. The ELISA procedures were carried out in accordance with the manufacturer's protocols to ensure standardization and accuracy. Each ELISA assay was repeated three times. The data were analyzed meticulously, and statistical significance was established at *p* < 0.05.

### 2.8 Quantitative Real‐Time PCR

The prostate tissues were thoroughly ground in liquid nitrogen, and subsequently total RNA was extracted utilizing the TRIzol reagent (Invitrogen, Carlsbad, CA, USA). The extracted RNA was subjected to reverse transcription using the PrimeScript RT reagent kit (Takara, Beijing, China). Real‐Time PCR was conducted on a 7500 System (Applied Biosystems Inc., Carlsbad, CA, USA) following a two‐step reaction procedure.

The expression of the housekeeping gene Gapdh was used to normalize the expression levels. The primers are designed to flank introns with Primer Premier 6.0 software (Premier Biosoft, Palo Alto, CA, USA). The melting curve checked the specificity of the primers. DNA sequencing and electrophoresis on the agarose gel have also tested the PCR products.

### 2.9 Western Blot

In brief, the prostate tissues were lysed using the RIPA lysis buffer; then the proteins were quantified using the BCA kit. The protein samples were loaded and separated through SDS‐polyacrylamide gel electrophoresis (SDS‐PAGE), transferred onto the polyvinylidene fluoride (PVDF) membranes, and blocked with 5% skim milk‐TBST for 2 h at 20°C. Then, the blots were incubated with primary antibodies (anti‐TFEB 1:500, Beclin‐1 1: 5000, anti‐p62/SQSTM1 1:2000, anti‐LC3 1: 2000, and anti‐GAPDH 1:20000, p‐AMPK 1: 2000, AMPK 1: 2000, p‐mTOR 1: 2000 and mTOR 1: 2000). After being washed and incubated with the secondary antibodies, chemiluminescence, X‐ray film compression, development, fixing, and data analysis were performed using Quantity One software (Bio‐Rad, CA, USA). The results represent three independent experiments.

### 2.10 Gut Microbiota Analysis

Fecal specimens were collected on the final day of the rat experiment and promptly subjected to rapid freezing using liquid nitrogen, then stored at −80°C for subsequent microbial community analysis. The intestinal content samples from four distinct rat groups were then utilized for 16S rDNA gene sequencing, with a sample size of *n* = 8 in each group. Genomic DNA was isolated from fecal samples of mice in accordance with the QiAamp Fast DNA Stool Mini Kit protocol. The amplification of 16S rDNA genes corresponding to the hypervariable region (16S V3–V4) was achieved through the utilization of a universal bacterial primer set, with Primer F = 341F (5′‐CCTACGGGNGGCWGCAG‐3′) and Primer *R* = 805R (5′‐GACTACHVGGGTATCTAATCC‐3′) (Shu et al. 2023). Subsequently, library preparation was conducted using the NEBNext Ultra II DNA Library Prep kit (New England Biolabs E7370S/L, Ipswich, MA, USA) and then sequenced on an Illumina MiSeq PE300 platform at Beijing Allwegene Technology Co. Ltd. (Beijing, China), resulting in the generation of 250 bp paired‐end reads. These reads were subsequently subjected to data processing steps, including merging, demultiplexing, and quality filtering using QIIME 2.0. Following these procedures, the reads were clustered into Operational Taxonomic Units (OTUs) at a 97% sequence identity threshold. All analytical outcomes were predicated on the characterization of OTUs. Based on OTU levels, alpha and beta diversity analyses were executed as per our prior study (Qiao, Xiao, et al. 2023). To pinpoint key OTUs responsive to distinct interventions, we employed linear discriminant analysis (LDA) coupled with effect size assessment. A *p*‐value of < 0.05 determined significance, and a minimum LDA score of ≥ 4.0 was deemed statistically significant.

### 2.11 Statistical Analysis

All data were presented as mean ± standard deviation (SD). Multiple comparisons were conducted using one‐way ANOVA test followed by Tukey's Honestly Significant Difference. A 95% confidence interval was used, and *p*‐values less than 0.05 were considered significant. All statistical analyses were performed using GraphPad Prism software version 10. The person who performed the procedures on the animals was different from the person who conducted group allocation and statistical analyses.

## 3 Results

### 3.1 RWPE‐1 Cell Viability

The impacts of six phenolamides (Figure 1A) with different concentrations (Figure 1B) on the viability of RWPE‐1 cells were evaluated using the MTT assay to determine the optimal dosage levels for subsequent investigations. As shown in Figure 1B, all phenolamides did not demonstrate significant growth‐inhibitory activities at a concentration of 20 μmol/L (*p* < 0.05), except P5 and P6. When the concentrations of P5 and P6 exceed 20 μmol/L (*p* < 0.05), RWPE‐1 cell survival was significantly decreased. Based on these results, concentration ranges (P1–P4 are 1, 5, 10, and 20 μM, P5 and P6 are 1, 5, and 10 μM) were chosen in the subsequent experiments.

### 3.2 Inflammatory Cytokines and NO Production

To assess the anti‐inflammatory effects of different phenolamides, we measured the levels of TNF‐α, IL‐1β, IL‐6, and NO production on LPS‐induced RWPE‐1 cells after being treated with various concentrations of phenolamides. As shown in Figure 1C, LPS dramatically increased the TNF‐α, IL‐6, IL‐1β, and NO levels. Phenolamides P1, P2, P3, and P4 demonstrated a concentration‐dependent reduction in the levels of inflammatory cytokines and NO, with the optimal at 20 μM. P5 and P6 exhibit anti‐inflammatory activity at their maximum non‐toxic concentration (10 μM). Noteworthily, P3 exhibited the most remarkable effects at 20 μM, with a respective decrease of 54.7%, 51.6%, 55.8%, and 67% in the levels of IL‐6, TNF‐α, IL‐1β, and NO, compared to the model group. The above results indicate that among the selected six phenolamides, P3 (N1(E), N10(E)‐di‐*p*‐coumaroyl spermidine, *p*‐Csp) exhibits a better anti‐inflammatory effect. Moreover, the anti‐inflammatory efficacy of P3 surpasses that of feruloyl putrescine (P1), which is the principal active constituent in the conventional pharmaceutical component known as Cernitin. Consequently, in subsequent investigations, we employ animal models to evaluate the anti‐prostatitis activity of *p*‐Csp.

### 3.3 Prostate Wet Weight and Prostate Index

**FIGURE 2:**
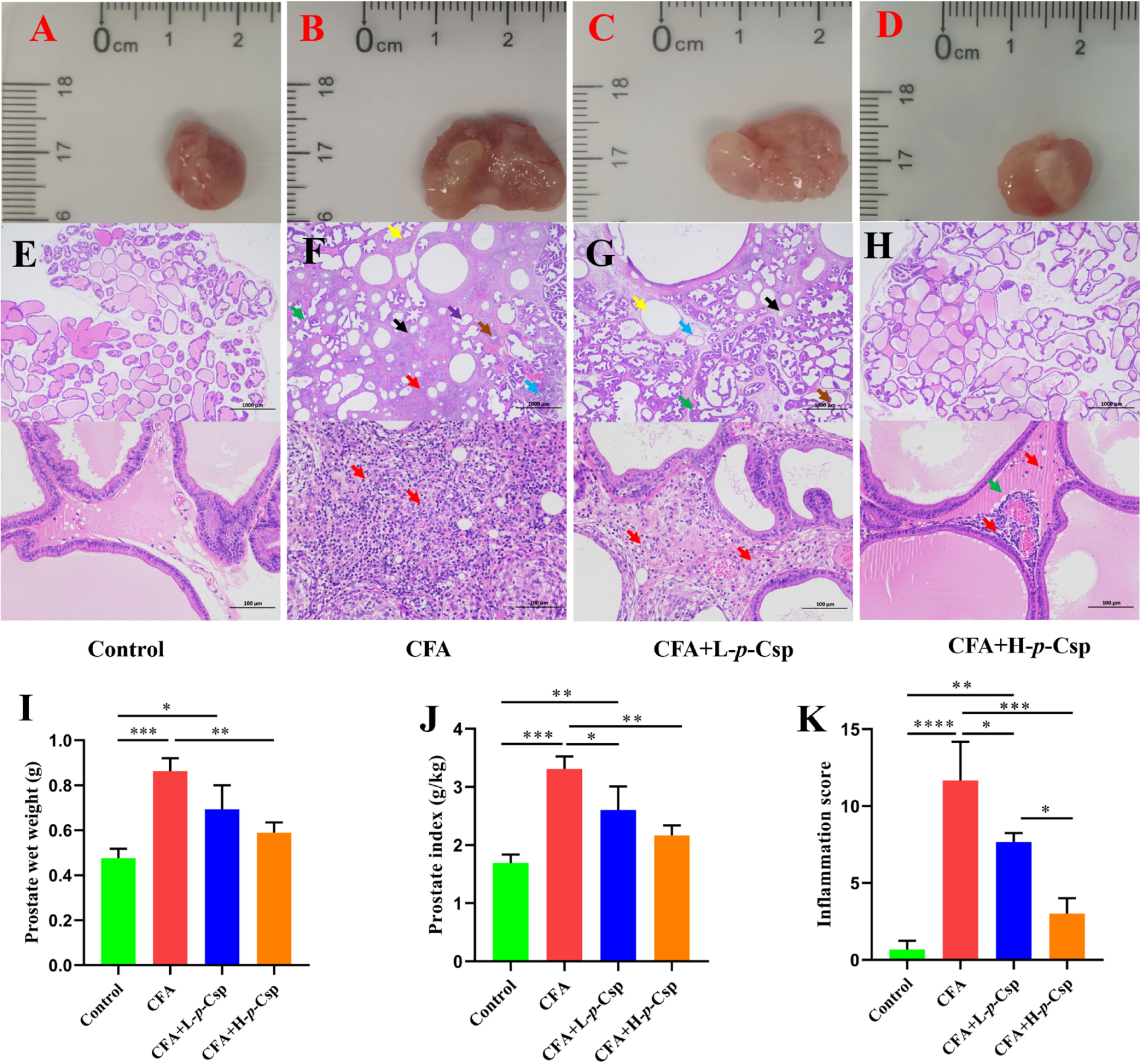
Effect of di‐*p*‐coumaroyl spermidine on prostate wet weight, prostate index, and histomorphometry in rats induced by CAF. (A–D) The morphology of prostate tissues; (E, F) The sections of rat prostates are stained with H&E (×100 and ×1000 magnification); (I) Prostate wet weight (g); (G) Prostate index (g/kg); (H) Inflammation score. *p < 0.05, **p < 0.01, ***p < 0.001, ****p < 0.0001.

Prostatomegaly is usually a common symptom in CNP. As shown in Figure 2A–D, CFA effectively induced the prostate extent in the prostatitis rat model. Compared to the control group (Figure 2A), the model group (Figure 2B) increased the prostate size. *p*‐Csp significantly decreased the prostate size and exhibited a concentration‐dependent response (Figure 2C,D), particularly the high dose group (Figure 2D) similar to the control group.

Prostate wet weight and the prostate index can quantitatively reflect the extent of prostate pathology (Qiao, Xiao, et al. 2023). In this study, a complete Freund's adjuvant‐induced model of nonbacterial prostatitis was used to evaluate the anti‐prostatitis effects of *p*‐Csp. CFA effectively induced a rat model of prostatitis, wherein the model group demonstrated a pronounced elevation in both prostate wet weight and prostate index, increasing 1.78 and 1.96‐fold, respectively, compared to the control group (Figure 2I,J). It is worth noting that *p*‐Csp significantly decreased both prostate wet weight and prostate index, exhibiting a concentration‐dependent response. In the high dose group (H‐*p*‐Csp), prostate wet weight and prostate index respectively reduced 31.6% and 34.4%, compared to the model group (Figure 2I,J).

### 3.4 Prostate Histomorphometry

Prostate histopathology can significantly display prostatitis lesions. To further evaluate the therapeutic effect of *p*‐Csp, prostatic histopathology was observed using the HE staining method. The model group (Figure 2F) reduced the number of glandular structures, along with connective tissue proliferation (black arrows), and formed numerous empty cavity structures (yellow arrows) and the infiltration of lymphocytes and granulocytes (red arrows), with a limited presence of inflammatory cells penetrating the glandular lumina (purple arrows). Multiple areas displayed signs of edema; connective tissue exhibited a loose arrangement (blue arrows). Eosinophilic material exudation was also observed (green arrows). Additionally, a significant reduction in secretions within many glandular lumina (brown arrows) and noticeable glandular expansion with thinning of glandular epithelium were noted. These evidences indicate the successful establishment of the CNP rat model (Figure 2F), compared to the control group (Figure 2E). After *p*‐Csp administration for 21 days, the glandular cavity structure of the prostate tissues recovered; the interstitial space, inflammatory cell infiltration in the glandular cavity and interstitial space, and migration of fibroblasts and blood vessels decreased dose‐dependent (Figure 2G,H). Specifically, the alleviation was most significant in the H‐*p*‐Csp (10 mg/kg) group, presenting a remarkable decrease in inflammatory cell infiltration and necrotic foci and lacking prostatic hyperplasia, resembling normal tissue (Figure 2H). Additionally, a comprehensive examination of the inflammation scores pertaining to histopathological alterations in the prostatic tissue was undertaken for each experimental group to assess the extent of inflammation (Figure 2K). Notably, compared to the model group, the groups receiving H‐*p*‐Csp treatment exhibited a significant reduction in inflammation scores, almost 67% (Figure 2K).

### 3.5 Immunohistochemistry

**FIGURE 3:**
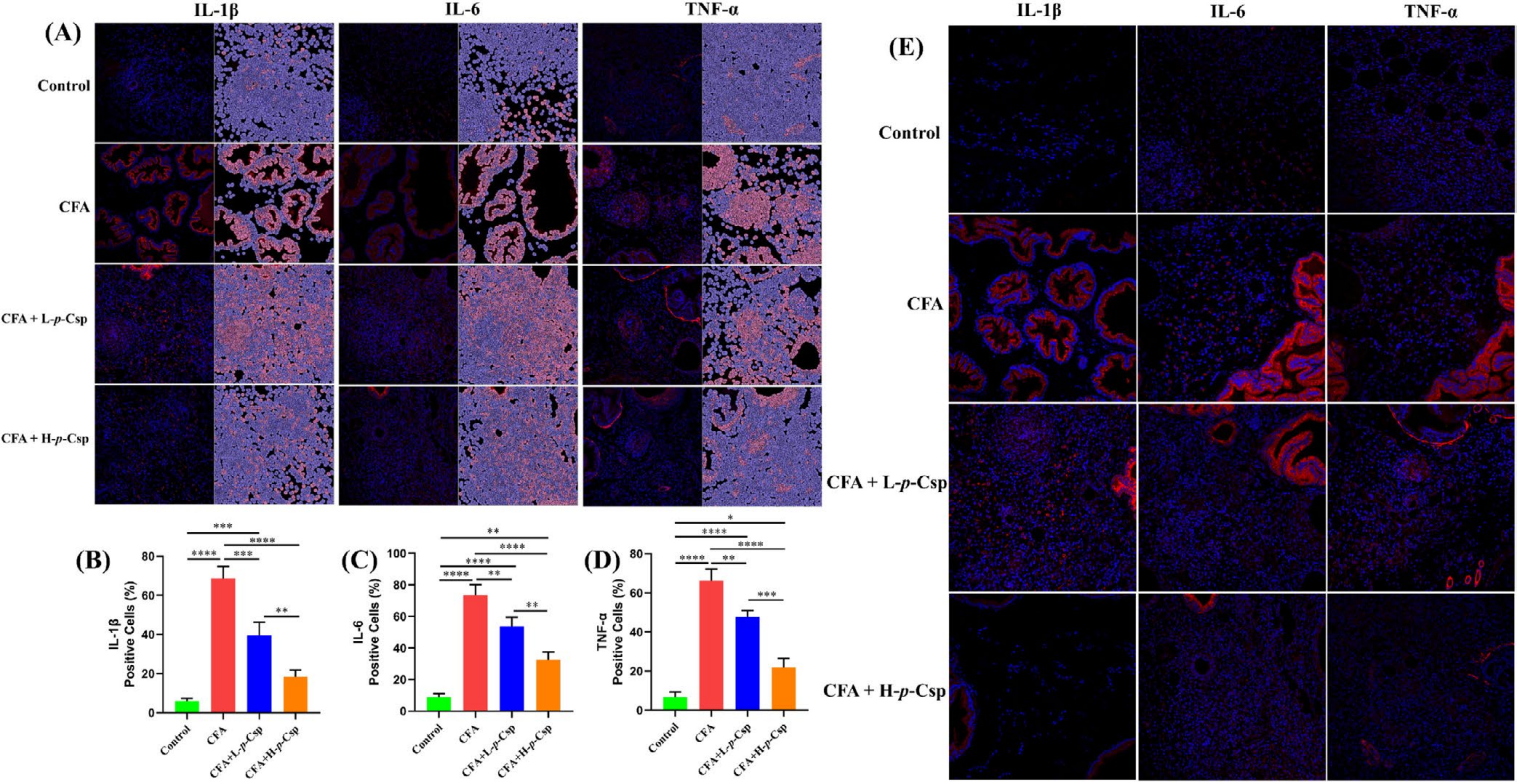
The expression analysis of proinflammatory cytokines using immunohistochemistry. (A) Immunochemical staining of IL‐1β, IL‐6, and TNF‐α in rat prostate tissue; (B–D) Positive cell rate of IL‐1β, IL‐6, and TNF‐α in rat prostate tissue. *p < 0.05, **p < 0.01, ***p < 0.001, ****p < 0.0001; (E) Confocal immunofluorescence imaging.

Immunohistochemistry was used to evaluate the expression of IL‐1β, IL‐6, and TNF‐α in prostate tissue (Figure 3A), and the positive cell ratio was calculated (Figure 3B–D). Confocal immunofluorescence imaging was used for the real‐time visualization analysis of prostate tissues (Figure 3E). As seen in Figure 3A, red fluorescence represented the inflammatory signal. The intensity increases of red fluorescence indicated a high expression of inflammatory cytokines, while blue fluorescence showed a normal cellular state. Based on the discernible increase in red fluorescence intensity, the model group (CFA treatment) heightened the expression of inflammatory factors (Figure 3A). Conversely, the two groups treated with *p*‐Csp were observed to reduce the levels of all three inflammatory factors and significantly exhibited a concentration‐dependent response (Figure 3E). By assessing the positive cell ratio, *p*‐Csp exerted a pronounced reversal effect on the elevated expression of IL‐6, IL‐8, and IL‐1β within the prostate tissue induced by CFA (Figure 3B–D). Noteworthily, the most substantial reduction was observed in the H‐*p*‐Csp group. IL‐1β notably decreased by approximately 69.8%, without a statistically significant difference between the H‐*p*‐Csp and control groups (Figure 3B). The effect of decreasing inflammatory factors in the H‐*p*‐Csp group was much better than that in the L‐*p*‐Csp group.

### 3.6 The Expression Levels of Inflammatory Cytokines in Tissue and Serum

**FIGURE 4:**
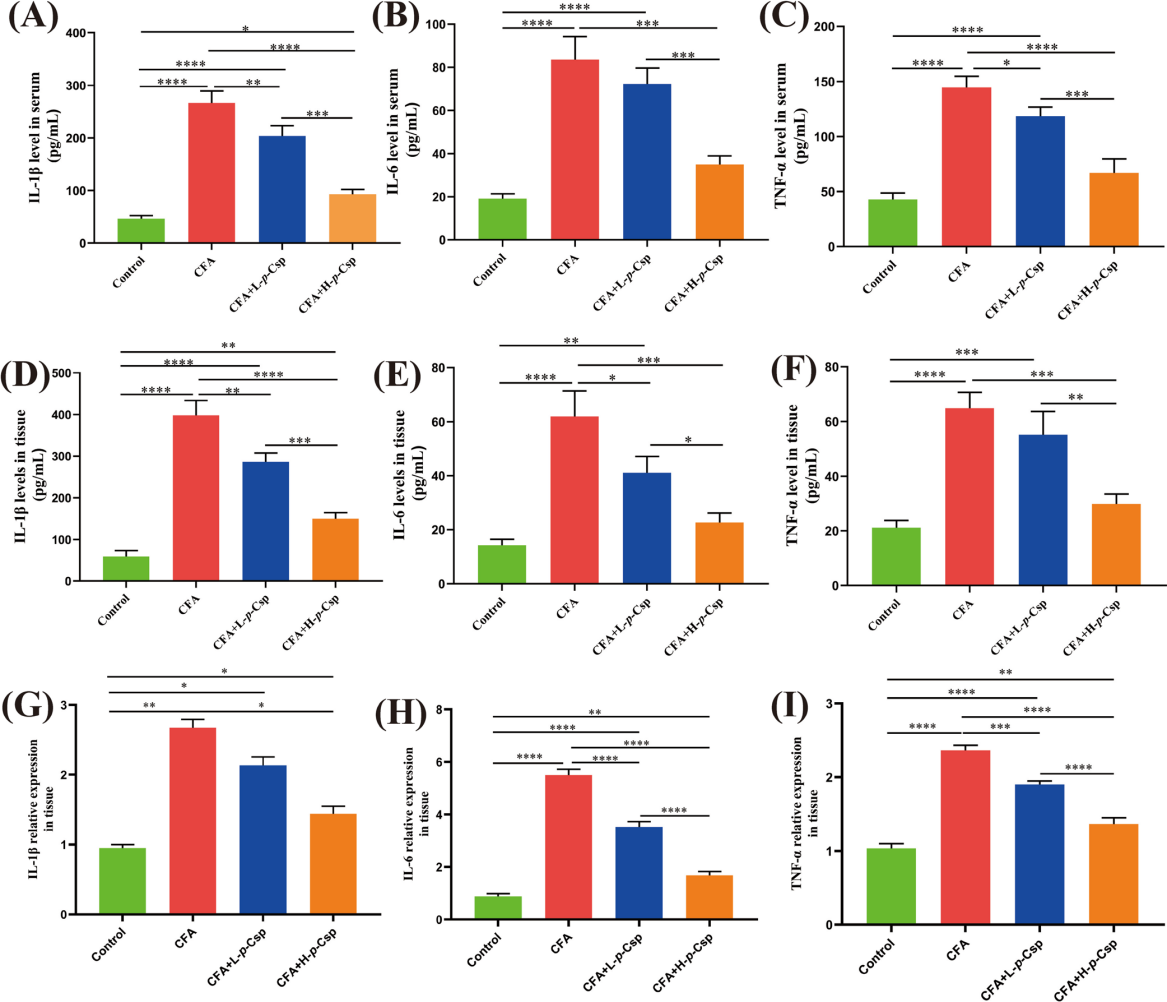
The expression levels of proinflammatory cytokines. (A–C) The expression levels of IL‐1β, IL‐6, and TNF‐α in rat serum; (D–F) the expression levels of IL‐1β, IL‐6, and TNF‐α in rat prostate tissue; (G–I) the expression levels of IL‐1β, IL‐6, and TNF‐α genes. *p < 0.05, **p < 0.01, ***p < 0.001, ****p < 0.0001.

CNP and prostate carcinoma patients are associated with prolonged overproduction of inflammatory cytokines, including IL‐1β, IL‐6, and TNF‐α. In this study, we determined the levels of IL‐1β, IL‐6, and TNF‐α in both serum and prostate tissue using ELISA kit. Figure 4 illustrates great variations in the levels of these inflammatory cytokines among different treatment groups. In contrast to the control group, the model group (CFA treatment) greatly elevated the levels of inflammatory cytokines in both blood (Figure 4A–C) and tissues (Figure 3D–F). Compared to the model group, two *p*‐Csp treatment groups conspicuously reduced inflammatory cytokine levels; notably, L‐6, IL‐1β, and TNF‐α levels in the H‐*p*‐Csp group decreased by approximately 50%. Additionally, gene expression profiles of proinflammatory cytokines IL‐1β, IL‐6, and TNF‐α were subjected to analysis (Figure 3G–I). In the model group, the genes responsible for encoding IL‐1β, IL‐6, and TNF‐α were conspicuously upregulated, compared to the control group. In contrast, the L‐*p*‐Csp and H‐*p*‐Csp groups exhibited marked down‐regulations of these gene expressions, compared to the model group. This further verified that *p*‐Csp treatment can conspicuously reduce inflammatory cytokine levels in both blood and prostate tissues.

### 3.7 Autophagy via the AMPK/mTOR Signaling Pathway

**FIGURE 5:**
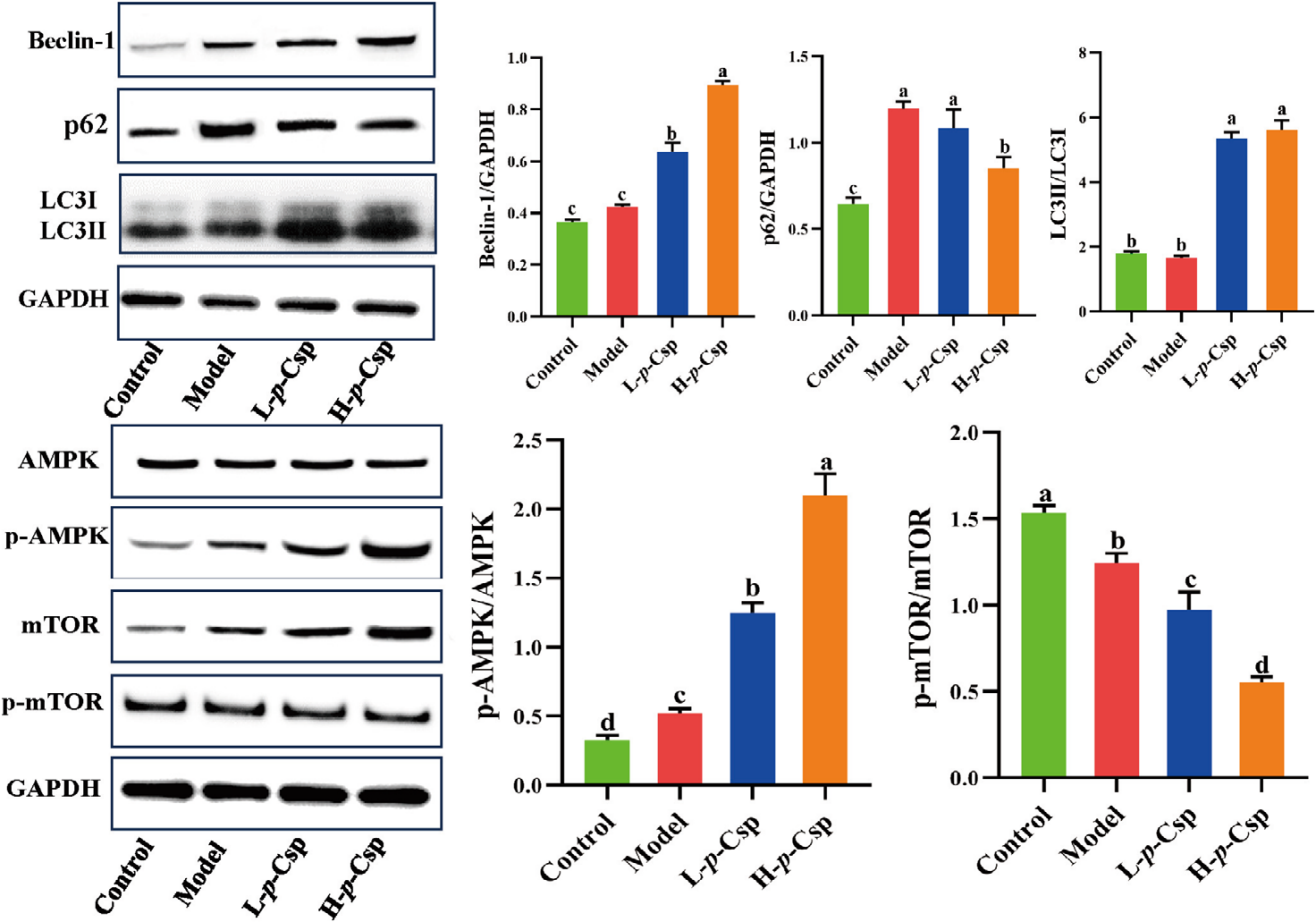
Di‐*p*‐Coumaroyl spermidine can upregulate autophagy via the AMPK/mTOR signaling pathway. Different letters (a, b, c and d) mean significant differences in other groups.

Autophagy plays a pivotal role in regulating inflammatory processes (Harris et al. 2017). In recent years, multiple studies have reviewed that activating cellular autophagy presents a promising therapeutic approach for managing chronic inflammation (Choi and Ryter 2011; Fan et al. 2015; Liu et al. 2019), particularly nonbacterial prostatitis (Su et al. 2018). The main proteins associated with autophagy include LC3 (microtubule‐associated protein 1A/1B‐light chain 3), Beclin‐1, and p62. To investigate the relationship between *p*‐Csp and autophagy, we detected the expression levels of autophagy‐related proteins by western blot analysis. As shown in Figure 5, the LC3‐II/LC3‐I ratio exhibited no notable alteration between the control and model groups. However, *p*‐Csp led to a significant increase in the LC3‐II/LC3‐I ratio. Moreover, the *p*‐Csp treatment group demonstrated a significant concentration‐dependent upregulation of Beclin‐1. Furthermore, p62 expression was decreased upon *p*‐Csp stimulation. Together, these results suggest that *p*‐Csp can enhance in vivo autophagy.

The AMPK‐mTOR pathway participates in the cellular response to energy deficiency, ultimately triggering autophagy (Hwang et al. 2021). Previous studies have offered compelling evidence supporting the notion that the activation of autophagy via the AMPK/MTOR signaling pathway holds significant potential for instigating anti‐inflammatory reactions (Fan et al. 2015; Hou et al. 2020; Su et al. 2018). To ascertain whether the role of *p*‐Csp in mitigating prostatitis is through activating autophagy by the modulation of the AMPK/MTOR signaling pathway, we detected the expression of AMPK/MTOR by western blot analysis. As seen in Figure 5, the *p*‐Csp treatment increased the ratio of p‐AMPK/AMPK, whereas the ratio of p‐mTOR/mTOR decreased. These findings imply that *p*‐Csp may potentiate autophagic processes through modulating AMPK and mTOR signaling pathways.

### 3.8 Gut Microbiota

Our previous research has proved that rapeseed bee pollen can mitigate CNP through regulating the gut microbiota (Qiao, Xiao, et al. 2023). To further investigate the potential role of *p*‐Csp, primarily found in rapeseed bee pollen, in gut microbiota, 16S rDNA amplicon sequencing analysis of rat feces was performed.

**FIGURE 6:**
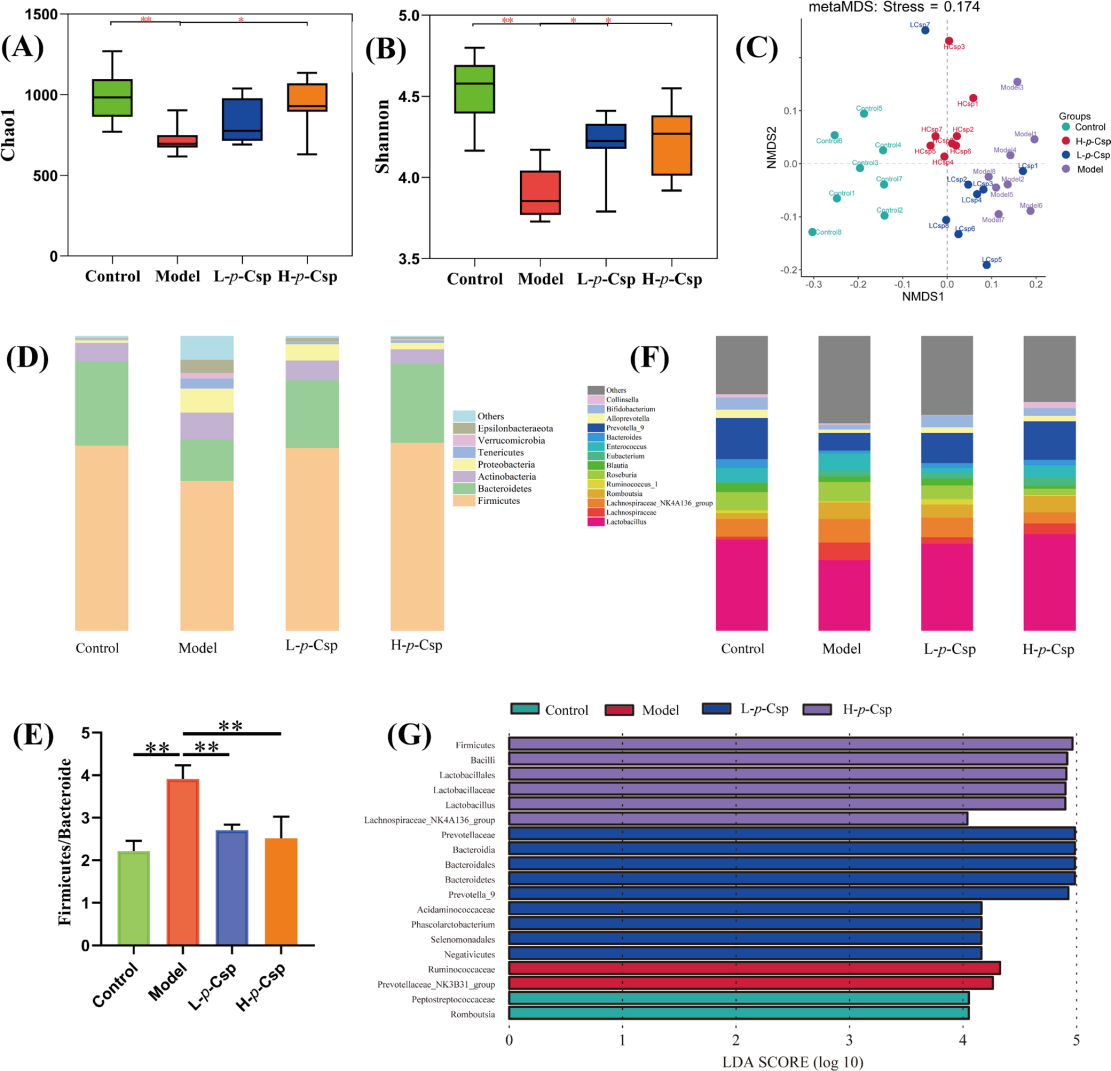
Effects of di‐*p*‐Coumaroyl spermidine on gut microbiota in CNP rat. (A) the Chao1 index; (B) Shannon index; (C) non‐metric multidimensional scaling (NMDS); (D) phylum level of gut microbiota; (E) genus level of gut microbiota; (F) the ratio of Firmicutes to Bacteroidetes; (G) linear discriminant analysis with LDA score greater than 4. Data are presented as mean ± SD (*n* = 8), **p < 0.01.

Chao1 and Shannon indexes expressed the alpha diversity. The Chao1 index estimates the total number of species in the sample, and the Shannon index describes the diversity of the microbial community. As shown in Figure 6A,B, alpha diversity with Chao1 and Shannon indexes on the OTU levels was significantly decreased in the model group compared to the control, suggesting that the structural diversity of gut microbiota decreased in the model group, while *p*‐Csp treatment reinstated alpha diversity. Additionally, non‐metric multidimensional scaling (NMDS) was employed as beta diversity assessment indexes to observe the structural variability of different species. As depicted in Figure 6C, the NMDS analysis unveiled noteworthy differences in the clustering patterns of gut microbiota species across the four experimental groups. A clear demarcation existed between the model and control groups; notably, the H‐*p*‐Csp group displayed more distinct segregation from the model group and closer to the control group. Hence, *p*‐Csp can change the gut microbiota structure, potentially contributing to mitigating CFA‐induced CNP.

To enhance our understanding of the bacterial taxa subject to regulation by *p*‐Csp, a comprehensive assessment of the gut microbiota composition was performed for the four experimental groups. The predominant bacterial phyla in all groups were Firmicutes, Bacteroidetes, Proteobacteria, and Actinobacteria (Figure 6D). In the model group, CFA treatment decreased the relative abundance of Bacteroidetes and increased the relative abundance of Firmicutes, thus significantly increasing the Firmicutes/Bacteroides (F/B) ratio. Noteworthily, *p*‐Csp treatment reduced the Firmicutes to Bacteroidetes ratio (F/B); for example, the H‐*p*‐Csp group is closer to the control group (Figure 6E). At the genus level (Figure 6F), compared to the control group, the model group decreased the relative abundance of Lactobacillus, Prevotella_9, Bifidobacterium, and Bacteroides, while increasing that of Lachnospiraceae, Romboutsia, and Lachnospiraceae_NK4A136_group. This finding suggested that *p*‐Csp can rectify the perturbations in gut microbial populations, with a particularly pronounced increase in Lactobacillus, Prevotella_9, Bifidobacterium, and Lachnospiraceae. Lactobacillus, Bifidobacterium, and Prevotella_9 are widely recognized as probiotic genera known to confer health benefits on host organisms. Moreover, LDA effect size (LEFSe) results showed that a total of 19 taxa at different OTU levels (LDA > 4) were identified with different abundances in the four groups (Figure 6G). The Negativicutes phylum was dominant in the model group, while Firmicutes were dominant bacteria in the model group, while Firmicutes were dominant bacteria in *p*‐Csp groups (Figure 6G). Moreover, Lactobacillaceae and Prevotella were obviously dominant in *p*‐Csp treatment groups. These results suggested that *p*‐Csp can modulate gut microbiota by increasing the abundance of beneficial bacteria in rats.

## 4 Discussion

Bee pollen and its extracts have been employed for over 40 years as dietary supplements or medication on a global scale in the therapeutic management of chronic prostatitis. However, it remains unclear which compounds in bee pollen are responsible for its anti‐chronic prostatitis effects and the underlying mechanisms, leading to product instability and lack of predictability. Recent studies have revealed that bee pollen is rich in phenolamides and is hailed as a treasure trove of phenolamides (Qiao, Feng, et al. 2023). Moreover, studies have already indicated that one of the components in the prostatitis treatment drug Cernitin is phenolamide. We speculated that the anti‐chronic prostatitis properties of bee pollen are mainly attributed to its richness of phenolamides. In this study, we evaluated the anti‐chronic prostatitis properties of six principal phenolamides in bee pollen based on cellular and rat models.

Our results indicate that the six phenolamides exhibited anti‐inflammatory activity in RWPE‐1 cells induced by LPS. Noteworthily, *p*‐Csp can remarkably decrease the levels of IL‐6, TNF‐α, IL‐1β, and NO, respectively (Figure 1C). The anti‐inflammatory efficacy of *p*‐Csp surpasses that of feruloyl putrescine, the principal active constituent (P1) in the conventional medicine Cernitin. Subsequently, *p*‐Csp also demonstrated a capacity effectively to in vivo mitigate CNP in the rat model, based on prostate wet weight, prostate index, and histomorphometry findings (Figure 2). *p*‐Csp treatments can decrease the levels and gene expression of inflammatory cytokines IL‐1β, IL‐6, and TNF‐α in serum and prostate tissue (Figure 4). These findings are consistent with the immunohistochemistry results (Figure 3). Our previous research has demonstrated that *p*‐Csp is a new found compound and is predominantly present in rapeseed bee pollen with a concentration of 10 mg/g [18]. Previous studies have consistently demonstrated that rapeseed bee pollen surpasses its counterparts in imparting anti‐prostatitis efficacy (Kujawski et al. 2015; Qiao, Feng, et al. 2023; Qiao, Xiao, et al. 2023). Our findings provide robust evidence that the efficacy of rapeseed bee pollen on prostatitis treatment may be mainly attributable to its high level of *p*‐Csp.

Phenolamides are formed by conjugating phenolic acids such as *p*‐coumaric acid, ferulic acid, and caffeic acid with polyamines (putrescine, spermidine, and spermine) through amide bonds. Research indicates that phenolamides present a variety of health benefits, such as anti‐inflammatory, antioxidant, and anti‐tyrosinase properties (Qiao, Feng, et al. 2023). *p*‐Csp is formed by two *p*‐coumaroyl residues bound at the N1 and N10 positions of spermidine. Spermidine has been reported to exert anti‐aging effects by stimulating autophagy and is hailed as an “anti‐aging vitamin” (Qiao, Feng, et al. 2023). As a solitary phenolamide in bee pollen, our results indicate that *p*‐Csp can activate the AMPK pathway, suppress the expression of mTOR, increase the LC3‐II/LC3‐I ratio, upregulate the expression of Beclin‐1, and downregulate the expression of p62, thereby inducing and promoting autophagy (Figure 5). Numerous studies have shown that autophagy is dependent on the regulation of the intracellular AMPK‐mTOR signaling pathway (Cao et al. 2021; Choi and Ryter 2011; Harris et al. 2017; Liu et al. 2019). AMPK exerts its inhibitory effect on mTOR by activating the TSC1/TSC2 complex (Tuberous Sclerosis Complex 1/2) (Hwang et al. 2021). The TSC1/TSC2 complex, by virtue of its capacity to suppress mTOR activity, facilitates the initiation of autophagy (Hindupur et al. 2015). Consequently, the activation of AMPK serves to repress mTOR, thus promoting the process of autophagy [33]. LC3, primarily in its cytosolic LC3‐I form, undergoes proteolytic cleavage to LC3‐II during autophagy induction (Cao et al. 2021). Elevated Beclin‐1 triggers autophagosome formation, while p62 accumulates during autophagy inhibition and decreases with active autophagy (Cao et al. 2021). Autophagy can attenuate inflammatory processes by facilitating the catabolism of proinflammatory cytokines and the clearance of compromised organelles, notably mitochondria, through a selective form of autophagy known as mitophagy (Choi and Ryter 2011; Hou et al. 2020). In recent years, studies have reviewed that activating cellular autophagy presents a promising therapeutic approach for managing chronic inflammation (Cao et al. 2021; Choi and Ryter 2011; Harris et al. 2017; Liu et al. 2019). Autophagy diminishes the biosynthesis of proinflammatory cytokines, potentially resolving the persistent inflammatory state emblematic of chronic prostatitis. Our results showed that the activating of autophagy by *p*‐Csp may be responsible for alleviating CNP (Figure 5). These results are consistent with the previously reported mechanism of rapamycin and IL‐37 exerting anti‐CNP and anti‐inflammation effects by activating autophagy through the AMPK/mTOR pathway (Fan et al. 2015; Hou et al. 2020). In this study, we first elucidated the mechanism against chronic prostatitis that phenolamides from bee pollen involve the activation of autophagy via the AMPK/mTOR pathway.

The gut microbiota is integral to the preservation of host health (Sekirov et al. 2010). Dysbiosis of the gut microbiota is associated with various inflammatory diseases, such as inflammatory bowel disease (Sultan et al. 2021), systemic inflammation in stroke patients (Yamashiro et al. 2017), and inflammatory conditions in diabetes patients (Wang et al. 2020). Dysbiosis involves decreased beneficial bacteria and increased opportunistic pathogens that stimulate proinflammatory mediator synthesis via plasma membrane receptor interaction (Qiao, Xiao, et al. 2023). Recent studies have revealed a correlation between dysbiosis of the gut microbiota and CNP (Qiao, Xiao, et al. 2023; White et al. 2021). Our results indicate that significant changes occur in the gut microbiota of CNP rats, consistent with those seen in CNP patients (White et al. 2021). *p*‐Csp treatment can significantly reduce the α‐diversity of the gut microbiota caused by prostatitis inflammation (Figure 6A,B). Some natural products have shown a similar trend in gut community diversity, including Puerarin and polysaccharides from the fruits of *Lycium barbarum* L. (Yang et al. 2022). Furthermore, *p*‐Csp can inhibit pathogenic bacteria and enhance probiotics. Compared to the model group, the H‐*p*‐Csp group significantly decreased the F/B ratio by upregulation of Bacteroidetes expression and downregulation of Firmicutes expression (Figure 6D). The F/B ratio was often used as an indicator to measure the health of intestinal flora; the high F/B ratio is closely related to inflammatory disease (W. Yang et al. 2022). Moreover, our results demonstrated that *p*‐Csp treatment can enrich the abundance of *Lactobacillaceae* and *Prevotella_9*, widely recognized as probiotic genera for host health. This is consistent with our previous report that rapeseed bee pollen can promote the growth of beneficial gut bacteria (Qiao, Xiao, et al. 2023). Our findings seem to provide a novel insight into modulating the gut microbiota with *p*‐Csp for preemptive intervention to forestall prostatitis, implicating the probable presence of a gut–prostate connection.

## 5 Conclusion

Our results reveal that phenolamides in bee pollen may be responsible for anti‐CNP. Specifically, N1(E), N10(E)‐di‐*p*‐coumaroyl spermidine can alleviate CNP by upregulating autophagy via the AMPK/mTOR signaling pathway and regulating gut microbiota. Based on our findings, we recommend that rapeseed bee pollen with a high level of N1(E), N10(E)‐di‐*p*‐coumaroyl spermidine should be selected to target prostatitis as nutraceuticals and therapeutics.

## Author Contributions

**Jiawen Zhang:** data curation (equal), formal analysis (equal), investigation (equal), validation (equal), visualization (lead), writing – original draft (equal). **Jiangtao Qiao:** conceptualization (equal), funding acquisition (equal), investigation (lead), methodology (lead), project administration (equal), resources (equal), software (equal), supervision (equal), writing – original draft (equal). **Yu Zhang:** investigation (equal), methodology (equal), resources (equal), software (equal). **Hequan Zhu:** investigation (equal), methodology (equal), resources (equal), software (equal). **Eric Haubruge:** validation (equal), writing – review and editing (lead). **Liqiang Liu:** conceptualization (equal), data curation (equal), investigation (equal). **Jie Dong:** funding acquisition (equal), project administration (equal), supervision (lead), writing – review and editing (equal).

## Ethics Statement

Animal care, breeding, handling, and experiments were approved by the Animal Welfare Committee of the Institute of Apicultural Research, Chinese Academy of Agricultural Sciences (Permit Number: 2022‐12; date of approval: 10 May 2022), in compliance with the Guide for the Care and Use of Laboratory Animals published by the NIH.

## Conflicts of Interest

The authors declare no conflicts of interest.

## Data Availability

The original data presented in this study are included in the article material. Further inquiries can be directed to the corresponding authors.

## References

[fsn370467-bib-0001] Algethami, J. S. , A. A. A. El‐Wahed , M. H. Elashal , et al. 2022. “Bee Pollen: Clinical Trials and Patent Applications.” Nutrients 14, no. 14: 2858. 35889814 10.3390/nu14142858 PMC9323277

[fsn370467-bib-0002] Campos, M. G. , S. Bogdanov , L. B. de Almeida‐Muradian , et al. 2008. “Pollen Composition and Standardisation of Analytical Methods.” Journal of Apicultural Research 47, no. 2: 154–161. 10.1080/00218839.2008.11101443.

[fsn370467-bib-0003] Cao, W. , J. Li , K. Yang , and D. Cao . 2021. “An Overview of Autophagy: Mechanism, Regulation and Research Progress.” Bulletin du Cancer 108, no. 3: 304–322. 10.1016/j.bulcan.2020.11.004. 33423775

[fsn370467-bib-0004] Chabot, S. , N. Dizeyi , L. Ramnemark , P. Lluel , P.‐A. Abrahamsson , and M. Grabe . 2021. “Impact of Cernitin on Induced Chronic Prostatitis in Animal Model for Understanding Management of Lower Urinary Tract Symptoms.” Phytomedicine Plus 1, no. 4: 100057. 10.1016/j.phyplu.2021.100057.

[fsn370467-bib-0005] Choi, A. J. , and S. W. Ryter . 2011. “Autophagy in Inflammatory Diseases.” International Journal of Cell Biology 2011: 2798. 10.1155/2011/732798. PMC3235460 22190939

[fsn370467-bib-0006] Chung, A. , M. Kini , S. M. Hartigan , B. Chughtai , and R. R. Dmochowski . 2021. “Nutritional Supplementation for Benign Prostatic Hyperplasia.” In Molecular Mechanisms of Nutritional Interventions and Supplements for the Management of Sexual Dysfunction and Benign Prostatic Hyperplasia, 107–111. Elsevier.

[fsn370467-bib-0007] Denisow, B. , and M. Denisow‐Pietrzyk . 2016. “Biological and Therapeutic Properties of Bee Pollen: A Review.” Journal of the Science of Food and Agriculture 96, no. 13: 4303–4309. 10.1002/jsfa.7729. 27013064

[fsn370467-bib-0008] El‐Khatib, F. M. , N. R. Yafi , and F. A. Yafi . 2019. “Over‐the‐Counter Supplements and Men's Health.” In Effects of Lifestyle on Men's Health, 281–300. Elsevier.

[fsn370467-bib-0009] Fan, X. , J. Wang , J. Hou , et al. 2015. “Berberine Alleviates Ox‐LDL Induced Inflammatory Factors by Up‐Regulation of Autophagy via AMPK/mTOR Signaling Pathway.” Journal of Translational Medicine 13: 1–11. 10.1186/s12967-015-0450-z. 25884210 PMC4365560

[fsn370467-bib-0010] Harris, J. , T. Lang , J. P. Thomas , M. B. Sukkar , N. R. Nabar , and J. H. Kehrl . 2017. “Autophagy and Inflammasomes.” Molecular Immunology 86: 10–15. 10.1016/j.molimm.2017.02.013. 28249679

[fsn370467-bib-0011] Hindupur, S. K. , A. González , and M. N. Hall . 2015. “The Opposing Actions of Target of Rapamycin and AMP‐Activated Protein Kinase in Cell Growth Control.” Cold Spring Harbor Perspectives in Biology 7, no. 8: a019141. 10.1101/cshperspect.a019141. 26238356 PMC4526743

[fsn370467-bib-0012] Hou, T. , X. Sun , J. Zhu , et al. 2020. “IL‐37 Ameliorating Allergic Inflammation in Atopic Dermatitis Through Regulating Microbiota and AMPK‐mTOR Signaling Pathway‐Modulated Autophagy Mechanism.” Frontiers in Immunology 11: 752. 10.3389/fimmu.2020.00752. 32411145 PMC7198885

[fsn370467-bib-0013] Hwang, H.‐Y. , J. S. Shim , D. Kim , and H. J. Kwon . 2021. “Antidepressant Drug Sertraline Modulates AMPK‐MTOR Signaling‐Mediated Autophagy via Targeting Mitochondrial VDAC1 Protein.” Autophagy 17, no. 10: 2783–2799. 10.1080/15548627.2020.1841953. 33124469 PMC8525979

[fsn370467-bib-0014] Kim, S. B. , Q. Liu , J. H. Ahn , et al. 2018. “Polyamine Derivatives From the Bee Pollen of *Quercus mongolica* With Tyrosinase Inhibitory Activity.” Bioorganic Chemistry 81: 127–133. 10.1016/j.bioorg.2018.08.014. 30118984

[fsn370467-bib-0015] Krieger, J. N. , D. E. Riley , P. Y. Cheah , M. L. Liong , and K. H. Yuen . 2003. “Epidemiology of Prostatitis: New Evidence for a World‐Wide Problem.” World Journal of Urology 21: 70–74. 10.1007/s00345-003-0329-0. 12712363

[fsn370467-bib-0016] Kujawski, R. , J. Baraniak , M. Kania , et al. 2015. “Diet Based on Oil of Seeds of *Brassica napus*. Implications for the Prevention and Treatment of Prostate Diseases.” Herba Polonica 60, no. 3: 77–88. 10.2478/hepo-2014-0018.

[fsn370467-bib-0017] Lazzarotto‐Figueiró, J. , A. Capelezzo , M. Schindler , et al. 2020. “Antioxidant Activity, Antibacterial and Inhibitory Effect of Intestinal Disaccharidases of Extracts Obtained From *Eugenia uniflora* L. Seeds.” Brazilian Journal of Biology 81: 291–300. 10.1590/1519-6984.224852. 32696852

[fsn370467-bib-0018] Liu, Y. , Y. Zhang , J. Peng , et al. 2019. “Autophagy Alleviates Ethanol‐Induced Memory Impairment in Association With Anti‐Apoptotic and Anti‐Inflammatory Pathways.” Brain, Behavior, and Immunity 82: 63–75. 10.1016/j.bbi.2019.07.033. 31376498

[fsn370467-bib-0019] Martinez‐Armenta, C. , M. C. Camacho‐Rea , G. A. Martínez‐Nava , et al. 2021. “Therapeutic Potential of Bioactive Compounds in Honey for Treating Osteoarthritis.” Frontiers in Pharmacology 12: 642836. 10.3389/fphar.2021.642836. 33967778 PMC8097136

[fsn370467-bib-0020] Münstedt, K. , and S. Bogdanov . 2009. “Bee Products and Their Potential Use in Modern Medicine.” Journal of ApiProduct and ApiMedical Science 1, no. 3: 57–63. 10.3896/IBRA.4.01.3.01.

[fsn370467-bib-0021] Nakase, K. , I. Kimura , and M. Kimura . 1990. “Effects of Pollen‐Extract Components, Diamines and Derivatives of Feruloylputrescine on Isolated Bladder and Urethral Smooth Muscles of Mice.” Japanese Journal of Pharmacology 53, no. 2: 157–164. 10.1254/jjp.53.157. 2385002

[fsn370467-bib-0022] Qiao, J. , Z. Feng , Y. Zhang , et al. 2023. “Phenolamide and Flavonoid Glycoside Profiles of 20 Types of Monofloral Bee Pollen.” Food Chemistry 405: 134800. 10.1016/j.foodchem.2022.134800. 36347200

[fsn370467-bib-0023] Qiao, J. , X. Xiao , K. Wang , E. Haubruge , J. Dong , and H. Zhang . 2023. “Rapeseed Bee Pollen Alleviates Chronic Non‐Bacterial Prostatitis via Regulating Gut Microbiota.” Journal of the Science of Food and Agriculture 103: 7896–7904. 10.1002/jsfa.12878. 37486857

[fsn370467-bib-0024] Sekirov, I. , S. L. Russell , L. C. M. Antunes , and B. B. Finlay . 2010. “Gut Microbiota in Health and Disease.” Physiological Reviews 90: 859–904. 10.1152/physrev.00045.2009. 20664075

[fsn370467-bib-0025] Shu, Y. , Y. Huang , W. Dong , et al. 2023. “The Polysaccharides From Auricularia Auricula Alleviate Non‐Alcoholic Fatty Liver Disease via Modulating Gut Microbiota and Bile Acids Metabolism.” International Journal of Biological Macromolecules 246: 125662. 10.1016/j.ijbiomac.2023.125662. 37399869

[fsn370467-bib-0026] Su, Y. , J. Lu , X. Chen , et al. 2018. “Rapamycin Alleviates Hormone Imbalance‐Induced Chronic Nonbacterial Inflammation in Rat Prostate Through Activating Autophagy via the mTOR/ULK1/ATG13 Signaling Pathway.” Inflammation 41: 1384–1395. 29675586 10.1007/s10753-018-0786-7

[fsn370467-bib-0027] Sultan, S. , M. El‐Mowafy , A. Elgaml , T. A. Ahmed , H. Hassan , and W. Mottawea . 2021. “Metabolic Influences of Gut Microbiota Dysbiosis on Inflammatory Bowel Disease.” Frontiers in Physiology 12: 1489. 10.3389/fphys.2021.715506. PMC8502967 34646151

[fsn370467-bib-0028] Thorpe, A. , and D. Neal . 2003. “Benign Prostatic Hyperplasia.” Lancet 361, no. 9366: 1359–1367. 10.1016/S0140-6736(03)13073-5. 12711484

[fsn370467-bib-0029] Wagner, A. E. , O. Will , C. Sturm , S. Lipinski , P. Rosenstiel , and G. Rimbach . 2013. “DSS‐Induced Acute Colitis in C57BL/6 Mice Is Mitigated by Sulforaphane Pre‐Treatment.” Journal of Nutritional Biochemistry 24, no. 12: 2085–2091. 10.1016/j.jnutbio.2013.07.009. 24231100

[fsn370467-bib-0030] Wang, G. , Q. Si , S. Yang , et al. 2020. “Lactic Acid Bacteria Reduce Diabetes Symptoms in Mice by Alleviating Gut Microbiota Dysbiosis and Inflammation in Different Manners.” Food & Function 11, no. 7: 5898–5914. 10.1039/C9FO02761K. 32572400

[fsn370467-bib-0031] White, B. , M. Welge , L. Auvil , et al. 2021. “Microbiota of Chronic Prostatitis/Chronic Pelvic Pain Syndrome Are Distinct From Interstitial Cystitis/Bladder Pain Syndrome.” medRxiv, 2021.2003. 2004.21252926. 10.1101/2021.03.04.21252926.

[fsn370467-bib-0032] Yamashiro, K. , R. Tanaka , T. Urabe , et al. 2017. “Gut Dysbiosis Is Associated With Metabolism and Systemic Inflammation in Patients With Ischemic Stroke.” PLoS One 12, no. 2: e0171521. 10.1371/journal.pone.0171521. 28166278 PMC5293236

[fsn370467-bib-0033] Yang, B. C. , L. L. Jin , Y. F. Yang , K. Li , and D. M. Peng . 2014. “Inhibitory Effect of Rape Pollen Supercritical CO_2_ Fluid Extract Against Testosterone‐Induced Benign Prostatic Hyperplasia in Rats.” Experimental and Therapeutic Medicine 8, no. 1: 31–37. 10.3892/etm.2014.1680. 24944593 PMC4061240

[fsn370467-bib-0034] Yang, W. , B. Gao , L. Qin , and X. Wang . 2022. “Puerarin Improves Skeletal Muscle Strength by Regulating Gut Microbiota in Young Adult Rats.” Journal of Orthopaedic Translation 35: 87–98. 10.1016/j.jot.2022.08.009. 36196075 PMC9508383

[fsn370467-bib-0035] Zhang, H. , X. Zhu , Q. Huang , et al. 2023. “Antioxidant and Anti‐Inflammatory Activities of Rape Bee Pollen After Fermentation and Their Correlation With Chemical Components by Ultra‐Performance Liquid Chromatography‐Quadrupole Time of Flight Mass Spectrometry‐Based Untargeted Metabolomics.” Food Chemistry 409: 135342. 10.1016/j.foodchem.2022.135342. 36586262

[fsn370467-bib-0036] Zhang, J. , C. Liang , X. Shang , and H. Li . 2020. “Chronic Prostatitis/Chronic Pelvic Pain Syndrome: A Disease or Symptom? Current Perspectives on Diagnosis, Treatment, and Prognosis.” American Journal of Men's Health 14, no. 1: 3200. 10.1177/155798832090320. PMC7256330 32005088

[fsn370467-bib-0037] Zhang, L. , Y. Wang , Z. Qin , et al. 2020. “Correlation Between Prostatitis, Benign Prostatic Hyperplasia and Prostate Cancer: A Systematic Review and Meta‐Analysis.” Journal of Cancer 11, no. 1: 177. 10.7150/jca.37235. 31892984 PMC6930406

[fsn370467-bib-0038] Zhang, X. , X. Wu , G. Xiao , et al. 2023. “Phenolamide Extract of Apricot Bee Pollen Alleviates Glucolipid Metabolic Disorders and Modulates the Gut Microbiota and Metabolites in High‐Fat Diet‐Induced Obese Mice.” Food & Function 14, no. 10: 4662–4680. 10.1039/D3FO01016C. 37102591

[fsn370467-bib-0039] Zhao, Q. , F. Yang , L. Meng , et al. 2020. “Lycopene Attenuates Chronic Prostatitis/Chronic Pelvic Pain Syndrome by Inhibiting Oxidative Stress and Inflammation via the Interaction of NF‐κB, MAPKs, and Nrf2 Signaling Pathways in Rats.” Andrology 8, no. 3: 747–755. 10.1111/andr.12747. 31880092 PMC7317562

